# Apolipoprotein A-I Supports MSCs Survival under Stress Conditions

**DOI:** 10.3390/ijms21114062

**Published:** 2020-06-05

**Authors:** Svetlana Miroshnichenko, Ivan Usynin, Alexey Dudarev, Vadim Nimaev, Anastasiya Solovieva

**Affiliations:** 1 Institute of Biochemistry, FRC FTM, 2 Timakova Street, 630630 Novosibirsk, Russia; ivan.usynin@niibch.ru (I.U.); alexdud@ngs.ru (A.D.); 2 Research Institute of Clinical and Experimental Lymphology—Branch of the ICG SB RAS, 2 Timakova Street, 630060 Novosibirsk, Russia; nimaev@gmail.com

**Keywords:** apolipoprotein A-I, mesenchymal stem/stromal cells, type 2 diabetes, MSCs transplantation, serum deprivation, hypoxia, oxidative stress, platelet lysate rich plasma

## Abstract

Clinical trials have shown the safety of mesenchymal stem/stromal cells (MSCs) transplantation, but the effectiveness of these treatments is limited. Since, transplanted MSCs will undergo metabolic disturbances in the bloodstream, we investigated the influence of blood plasmas of type 2 diabetes (T2D) patients on MSCs viability and examined whether apolipoprotein A-I (apoA-I) could protect cells from stressful conditions of serum deprivation (SD), hypoxia, and elevated concentrations of reactive oxygen species (ROS). ApoA-I exhibits anti-inflammatory, immune activities, improves glycemic control, and is suitable for T2D patients but its influence on MSCs remains unknown. For the first time we have shown that apoA-I decreases intracellular ROS and supports proliferative rate of MSCs, thereby increasing cell count in oxidation conditions. ApoA-I did not influence cell cycle when MSCs were predominantly in the G0/G1 phases under conditions of SD/hypoxia, activated proliferation rapidly, and reduced apoptosis during MSCs transition to the oxygenation or oxidation conditions. Finally, it was found that the blood plasma of T2D individuals had a cytotoxic effect on MSCs in 39% of cases and had a wide variability of antioxidant properties. ApoA-I protects cells under all adverse conditions and can increase the efficiency of MSCs transplantation in T2D patients.

## 1. Introduction

Nowadays, mesenchymal stem/stromal cells (MSCs) have become an attractive tool for regenerative medicine due to their self-renewal, multilineage, and immunosuppressive capacities as well as the ease of their isolation by standard methods. Their capability to migrate and repair injured tissues and organs makes them a very promising tool for transplantation.

Thanks to these properties, MSCs have been introduced in clinical trials for bone and cartilage regeneration [1,2] and the treatment of immune disorders [3,4], critical limb ischemia [5], bronchopulmonary dysplasia [6], type 2 diabetes (T2D) [7,8,9], type 1 diabetes [10], and heart disease [11,12]. MSCs are emerging as an extremely promising therapeutic agent for regeneration thanks to their ability to engraft and differentiate into different kinds of cells and mediate paracrine effects by a wide range of secreted soluble factors [13,14,15,16]. The safety of introduced MSCs has been shown in several clinical trials but the effectiveness of treatment was limited owing to cell death during transplantation due to the harsh microenvironment within damaged recipients tissues [17,18,19].

MSCs have of the unique ability to modulate the immune response at the level of both innate and adaptive immunity and, due to these properties, are considered to be most suitable cells for the treatment of atherosclerosis and T2D [16]. However, studies have shown heterogeneous results when MSCs are used to treat these diseases [20,21].

It was shown that metabolic dysregulation and the development of oxidative-antioxidant systems dysfunction negatively affect stem cells, causing their aging and apoptosis in T2D patients [22,23,24]. Most likely, such an environment in the recipient will affect transplanted MSCs in the same way, reducing the efficacy of cell therapy.

Hyperglycemia, insulin resistance (IR), the disruption of insulin synthesis by β cells supports a state of chronic inflammation in T2D patients. These conditions are accompanied by elevated reactive oxygen species (ROS) levels and the accumulation of lipid peroxidation toxic products [20,25,26], as well as a reduction of apolipoprotein A-I/high-density lipoprotein (apoA-I/HDL) levels, including the appearance of a modified protein and dysfunctional apoA-I/HDL particles [27,28,29]. In turn, this leads to a multitude of vascular complications in T2D patients, including coronary artery disease, myocardial infarctions, cerebrovascular disease, peripheral arterial disease, and atherosclerosis [20,30,31,32]. Recent clinical studies demonstrate that rheumatoid arthritis (RA) is associated with the same risk factors as T2D, which is complicated by accelerated atherosclerosis, leading to the increased risk of cardiovascular disease (CVD) [33,34]. Cell therapy has shown its advantages and shortcomings, demonstrating efficacy in 40% of T2D patients only [9,20]. Hence, there is currently interest in the search for new approaches to increase the efficacy of cell therapy. Previous research has demonstrated that apoA-I exhibits a glucose controlling function at the expense of lowering the insulin resistance, strengthening the absorption of glucose by cells in an insulin-dependent and insulin-independent manner [35,36].

ApoA-I is the main protein component of HDL particles and responsible for their main functions. ApoA-I/HDL binds and reverses cholesterol transport, providing an anti-sclerotic, cardioprotective effect, and also exhibits anti-inflammatory, anti-apoptotic, and immune activities [37,38,39,40]. This protein is actively involved in the anti-inflammatory strategies of the body, and in an remarkable way, involved in the regulation of both innate and adaptive immunity [39,41,42,43].

It was shown that apoA-I influences the functional activity of cells to have a protective effect on endothelial cells by inhibiting apoptosis and increasing cell proliferation [44]. We have previously shown that apoA-I stimulates the proliferation of granulocytic and monocytic cells in rat bone marrow in conditions of serum deprivation [45]. The proliferation rate of MSCs is an essential parameter for stem cell therapeutic interventions and so it has been of great interest to protect MSCs from elevated ROS and to support cell proliferation in dysfunctional metabolic environment.

Recently emphasized the importance of lipids are active players in signaling of the key metabolic processes. One of the most important reactions of lipids is the autocatalysed chain reaction of peroxidation [25]. ApoA-I binds single-electron oxidants and reduces the amount of lipid hydroperoxides (LOOH) by the formation of redox-inactive lipid hydroxides (LOH). Thereby, the termination of chain reactions of lipid peroxidation and the redox state of apoA-I Met residues (the positions 112 and 148) and Tyr 115 are essential for HDL antioxidative capacity, following the transfer of LOOH from low density lipoprotein (LDL) particles to HDL [26].

Having all of the above properties, apoA-I is a promising candidate for both the inclusion in serum-free media for MSCs propagation and as a therapeutic agent for transplantation of MSCs to treat atherosclerosis-associated disease, such as CVD, myocardial infarction, diabetes, metabolic syndrome, and rheumatoid arthritis.

Indeed, one of the approaches for the correction of lipid metabolism disorders accompanying all these diseases is the introduction of a native protein, as well as mimetics of α-helical regions of apoA-I. These apoA-I mimetics were investigated as a therapeutic agent of cardiovascular diseases [46]. The potential of the use of apoA-I in atherosclerosis [47] and metabolic syndrome [48] were also shown.

Probably, the use of apoA-I protein, which has glucose-regulating function and affects the components of innate and adaptive immunity, together with MSCs that have similar immunomodulatory functions, has great potential for the development of regulatory cell-mediated approaches for the treatment of T2D and its complications. Therefore, the aim of this study was to investigate the influence of apoA-I on MSCs survival and proliferative rate under harmful conditions (nutritional starvation by serum deprivation, hypoxic conditional, and oxygen stress by H_2_O_2_). Investigations were performed with the aim of understanding possible negative effects of plasma of T2D patients on the survival of MSCs and a possible protective effect of doping plasma of patients with apoA-I.

## 2. Results

### 2.1. Analysis of apoA-I Secondary Structure Reveals a High Content of α-Helices

**Figure 1:**
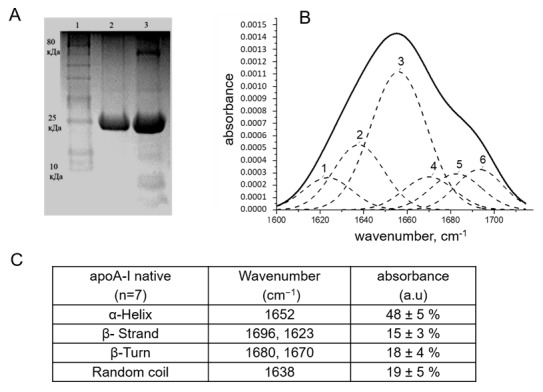
Characterization of the apolipoprotein A-I (apoA-I). (**A**) The protein purity: SDS-PAGE analysis. 1: standard molecular weight marker (10–80 kDa), 2: apoA-I, 3: High-density lipoprotein (HDL). (**B**) Fourier Transform Infrared Spectroscopy (FTIR) analysis of apoA-I secondary structure. Peak deconvolution of Amide I of apoA-I using OriginPro software. Peak 3—α-Helix; peaks 1, 6—β-Strand; peaks 4, 5—β-Turn; peak 2—random coil. (**C**) The secondary structure content (%) of apoA-I.

For an adequate investigation of the function of apoA-I, it is necessary to demonstrate that the protein used in investigations has retained its native structure unchanged. The extraction of apoA-I from blood plasma by fractionation of HDL, delipidation under non-denaturing conditions, with a mixture of butanol-isopropyl ether and gel filtration gives a protein with a purity of approximately 95% (Figure 1A) with a native structure, which is consistent with the further characterization of its secondary structure (Figure 1). It was shown that the predominant secondary structural feature of the obtained protein is a high content of amino acid segments forming α-helices when they were analyzed by Fourier Transform Infrared Spectroscopy (FTIR). The band situated at 1600–1700 cm^−1^ (Amide I band) is the most frequently studied to analyze the secondary structure of proteins by FTIR spectroscopy. The Figure 1C presented the percentage of structural elements of the Amide I band. The dashed “Gaussians” in Figure 1B correspond to the profile of the structural elements of the Amide I band, the profile of which is represented by a solid line. Peak 3 (1652 cm^−1^) belongs to α-helices and is predominant according to the results of deconvolution of the infrared spectrum and the second derivative. Thus, the content of α-helices in apoA-I has a value from 48% to 53%. The high content of α-helical regions is important for the manifestation of the basic properties of apoA-I/HDL. Thus, we obtained a protein of conformational preservation of the native protein.

### 2.2. ApoA-I Did Not Increase the Proliferation and Nuclease Activity of MSCs in a Microenvironment Mimicking the Stem Cell Niche

#### 2.2.1. Viability Cell Assay and Cell Cycle Analysis

**Figure 2:**
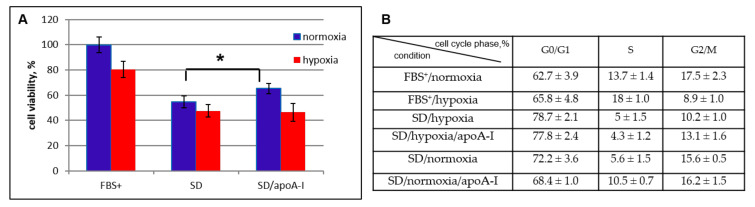
The effect of apoA-I on (**A**) the viability of bone marrow mesenchymal stem/stromal cells (MSCs) under conditions: with fetal bovine serum (FBS^+^)/hypoxia (5% O_2_); FBS^+^/normoxia (20% O_2_); serum deprivation (SD)/hypoxia; SD/normoxia and SD/hypoxia/apoA-I, SD/normoxia/apoA-I and (**B**) cell cycle of bone marrow MSCs under the same conditions assayed by flow cytometry analysis. * *p* < 0.05.

The majority of investigations describes the effects of HDL particles, but there is no data describing the influence of apoA-I on functional activity of MSCs. The transplantation of MSCs is used in various pathological conditions. Mostly, those conditions are characterized by the presence of oxidative stress, hypoxia, and nutrient deficiency resulting from the disruption of the microcirculation. To investigate the influence apoA-I on the biological activity of MSCs, we performed an MTT (3-(4,5-dimethylthiazol-2-yl)-2,5-diphenyltetrazolium bromide) tetrazolium assay and flow cytometry analysis to detect cell viability under stress conditions of serum and oxygen deprivation. Serum deprivation (SD) under normoxia conditions (21% O_2_, norm O_2_) reduces the percentage of living cells to 54 ± 4.5% compared to cultivation in a complete medium (cell survival in complete medium is taken as 100%). The addition of apoA-I into culture medium increases MSCs viability by 10 ± 2% (Figure 2A, blue column), and 67 ± 6% of the cells were viable under hypoxia (5% O_2_). Additional serum deprivation under hypoxia reduced cell survival to 48%, the addition of apoA-I did not affect this indicator (Figure 2A, red column). To understand the decrease of cell viability under SD/hypoxic condition assayed by MTT we analyze the cell cycle of MSCs under the same conditions. The cell cycle analysis of MSCs (passages 5–8) cultivated under hypoxia and serum deprivation conditions showed decreased levels of proliferative activity, two times than under the standard cultivation conditions (S phase 5% and 13.7% respectively, Figure 2B). Under these conditions, apoA-I does not increase the rate of cell proliferation (S phase 4.3%) (Figure 2A,B).

SD/normoxia conditions led to the elevation of G2/M phases relatively hypoxic conditions. ApoA-I addition elevates S phase 1.9 times than SD/normoxia conditions (Figure 2B). The apoptosis was not detected by cell cycle analysis and caspase 3.7 assay (data not shown) in SD/hypoxia condition.

Thus, a decrease of MSCs survival up to 48%, estimated by the MTT test, is associated with a decrease of cell proliferative activity, as shown by flow cytometry analysis under conditions of serum and oxygen deprivation.

#### 2.2.2. The Nuclease Activity Assay

All DNA remodeling processes are associated with nuclease activity, as nucleases participate in almost all vital processes in the cell: completing apoptosis (nucleosomal degradation), deoxyribonucleic acid (DNA) replication, recombination, and repair. Accordingly, the re-entry of MSCs into the quiescent state will be accompanied by a decrease of nuclease activity level. The study covered the total pool of magnesium-dependent alkaline (pH 8.3), neutral (pH 7.2), and acidic (pH 5) nucleases.

**Figure 3:**
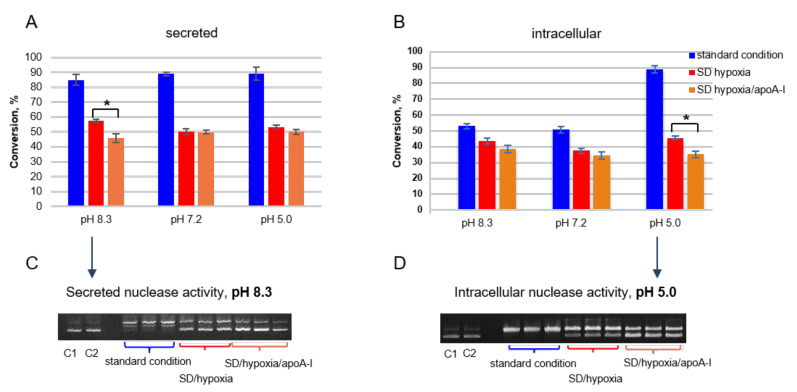
Percent of conversion of supercoiled plasmid DNA (pDNA Bluescript) into nicked species. Nuclease activity estimated from (**A**) homogenized MSCs (intracellular activity) and (**B**) culture medium (secreted activity). * *p* ≤ 0.01. (**C**) 0.8% agarose gel illustrating the secreted nuclease activity, pH 8.3. (**D**) 0.8% agarose gel illustrating the intracellular nuclease activity, pH 5.0. C1—control 1-whole pDNA, C2—control 2-pDNA/cells (for intracellular) or media (for secreted)/EDTA. Samples were studied in triplicate. Full gels electrophoregram is presented in Figure S1 (see Supplementary Materials).

Under standard culture conditions, the activity of secreted nucleases was equal to 88 ± 2% plasmid DNA (pDNA) hydrolysis at all pH values (Figure 3A, blue column). The activity of secreted nucleases was significantly decreased at all pH values (39 ± 3.3%) under serum and oxygen deprivation conditions (Figure 3A, red column). The addition of apoA-I (orange column) into culture media reduced the activity of secreted alkaline nucleases by 11.5% (*p* < 0.01) (Figure 3A,C). ApoA-I did not influence secreted nuclease activity at pH 7.2 and pH 5.0 (Figure 3A and Figure S1).

The activity of intracellular alkaline and neutral nuclease decreased by 9.4% and 13.3% under SD/hypoxia conditions respectively (Figure 3B, red column compared blue column). The maximum decrease in activity (by 43.4%) was noted for intracellular acid nucleases. ApoA-I reduced the activity of intracellular acid nucleases by 10.5% additionally (*p* < 0.01) (Figure 3B,D) and did not significantly affect other pH-dependent nucleases.

The down regulation of nuclease activity, which was stronger in the case of secreted nucleases, is in good agreement with lowering all processes in MSCs when they are in a dormant state.

### 2.3. ApoA-I Enhance Proliferation Rate of MSCs under Serum Deprivation Condition

#### 3H-Thymidine Incorporation Assay

To understand the effect of apoA-I on the viability of bone marrow mesenchymal cells under SD conditions better, the level of proliferative activity of the cells was determined by the incorporation of 3H-thymidine depending on the time of incubation with the protein.

**Figure 4:**
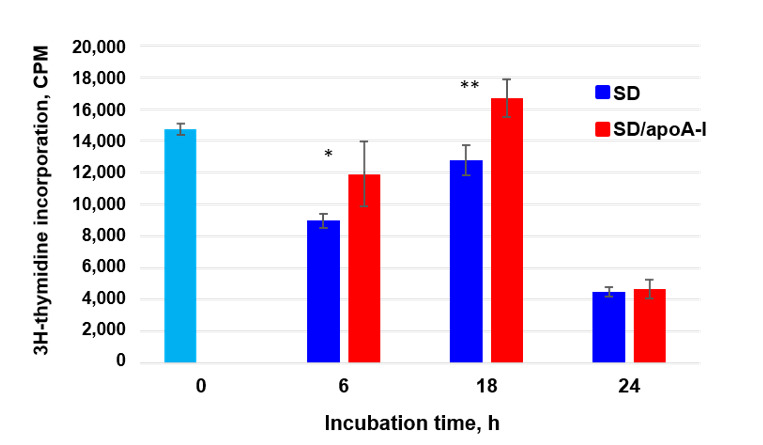
Effect of apoA-I on MSCs proliferation under condition of serum deprivation (SD) analyzed by 3H-thymidine incorporation assay. Light blue column—3H-thymidine incorporation under standard cultivation conditions (FBS^+^). Data was shown as counts per minute (CPM) per well. * *p* ≤ 0.05, ** *p* ≤ 0.01 compared to SD and SD/apoA-I conditions.

Serum deprivation reduced the rate of 3H-thymidine incorporation compared with cultivation in a complete nutrient medium (Figure 4, light blue column). Doping the serum-free medium with apoA-I maintained the proliferative level of MSCs, which was significantly higher than under SD conditions by 32.9 ± 4.8% after 6 h of incubation and by 31.2 ± 4% after 18 h (Figure 4). After 24 h of incubation of cells with apoA-I, there was no significant difference in the number of counts per minute (CPM).

### 2.4. ApoA-I Enhances MSCs Viability in the Model of Oxidative Stress by Down Regulation of Intracellular Reactive Oxygen Species

#### 2.4.1. Cell Cycler Analysis

The high glucose and insulin blood levels induced high intracellular ROS levels and led to stem cell aging and apoptosis. Accordingly, we investigated whether apoA-I is protecting MSCs against oxidative stress-induced apoptosis. We used 100 and 500 μM of H_2_O_2_ to modulate oxidative stress.

**Figure 5:**
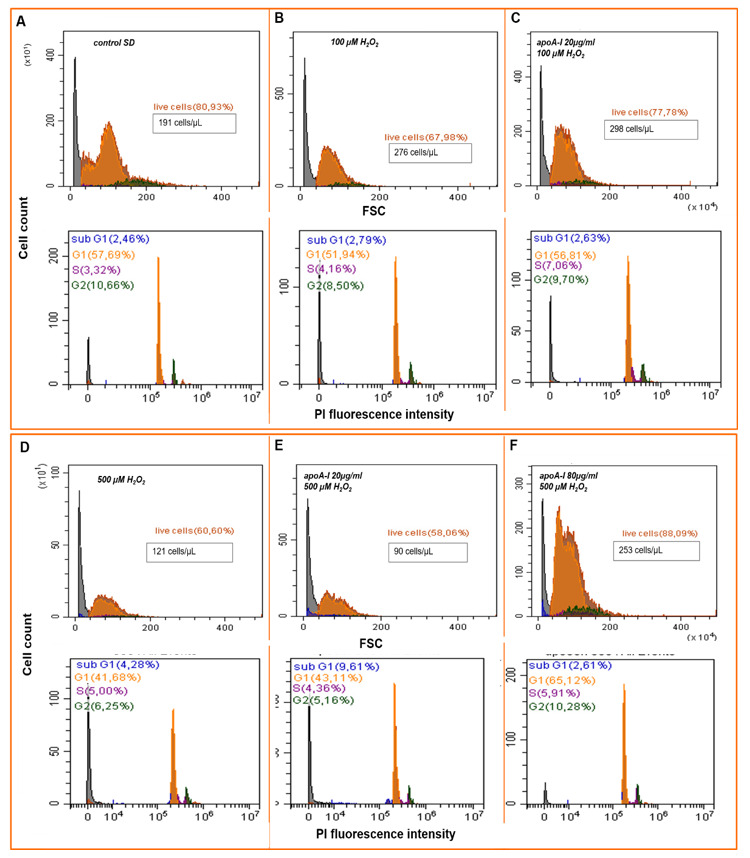
ApoA-I supports MSCs proliferative activity and decreases cell death under peroxidation conditions. (**A**–**C**) show representative flow cytometry assays of MSCs viability under oxidation by 100 µM H_2_O_2_. Histograms: A—control SD condition, B—100 µM H_2_O_2_, C—preconditioning of MSCs by 20 µg/mL apoA-I followed by adding 100 µM H_2_O_2_. (**D**–**F**) show representative flow cytometry assays of MSCs viability under oxidation by 500 µM H_2_O_2_. Histograms: D—500 µM H_2_O_2_, E—preconditioning of MSCs by 20 µg/mL apoA-I followed by adding 500 µM H_2_O_2_, F—preconditioning of MSCs by 80 µg/mL apoA-I followed by adding 500 µM H_2_O_2._ Upper panels of (**A**–**F**) are forward side scatter (FSC) histograms of MSCs content, dead and live cells; bottom panels are FACS histograms of MSCs DNA content reflect cell cycler phases by Propidium Iodite (PI) fluorescent intensity.

Through cell cycle analysis, we observed an increased percentage of cells in the gate of living cells when MSCs were incubated in the presence of apoA-I in the culture medium under oxidative stress conditions (Figure 5C,F upper panels).

The proliferation rate of MSCs under SD/normoxia conditions was S phase 3.5 ± 1.1% and G2/M 12.3 ± 1.5% (Figure 5A). The addition of 100 μM of H_2_O_2_ to culture medium causes an increase in the cell count but also an increase of the dead cell content (30.1 ± 2.2%) (Figure 5B). ApoA-I increases the cell content and S phase to 7.3 ± 2.3 %, reducing the dead cell content to 21 ± 0.9% (Figure 5C). In this way, the addition of 20 µg/mL protein to the culture medium was enough to support the viability of MSCs under the effect of 100 μM H_2_O_2_.

It was shown that 500 μM H_2_O_2_ are toxic for MSCs, causing cell death (41.5% dead cells and 3.7 ± 1.2% apoptotic cells) (Figure 5D). Hydrogen peroxide activates cell proliferation, increasing the S phase relative to the control (SD/normoxia) by a factor of 1.5. Adding of apoA-I at a concentration of 20 µg/mL did not protect cells from oxidation damage. Hence, apoptotic events increased to 9.5 ± 1.9%, and among dead cells was 43.1 ± 2.1% (Figure 5E).

Elevated apoA-I concentration in culture medium to 80 μg/mL significantly reduces necrosis to 12.2 ± 0.9% and apoptosis to 2.6 ± 0.6% and maintains the high proliferation level, thereby increasing the total number of cells by 2.1 times in 5 h of incubation (Figure 5F).

Therefore, it was shown that in order to increase the viability of cells incubated at high peroxide concentrations, a correspondingly higher apoA-I content is required.

#### 2.4.2. Detection of Intracellular ROS Species

To explore the mechanism of apoA-I protective influence on MSCs viability under conditions of oxidative damage, we determined intracellular ROS content after cell treated 100 and 500 μM H_2_O_2_.

**Figure 6:**
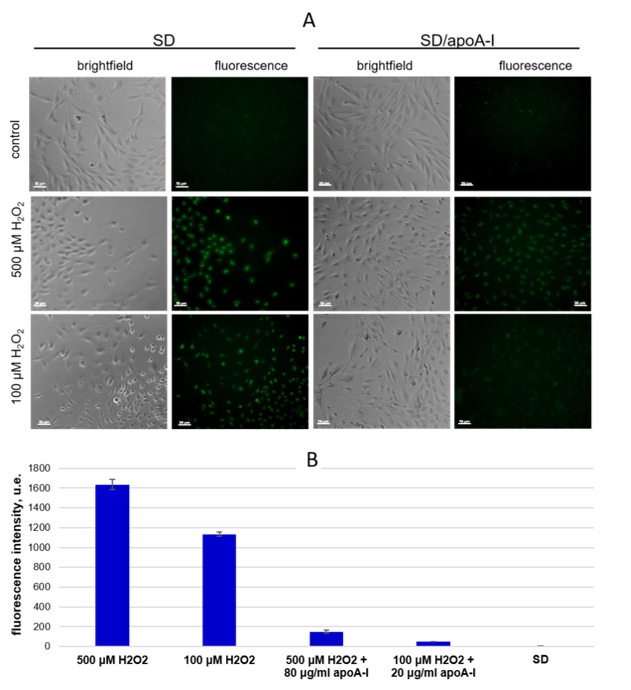
ApoA-I reduces intracellular ROS species was induced by 100 μM and 500 μM H_2_O_2_. CellROX^®^ Green reagent was used as an intracellular reactive oxygen species (ROS) detector. (**A**) Brightfield and green fluorescent channel micrographs of MSCs, ×20, scale bar 50 µm. (**B**) Fluorescence Intensity calculated using software Cell Activision R1.03.01. The results are presented as arbitrary fluorescence units (u.e.).

The addition of peroxide (100 and 500 μM) causes a cell volume reduction, reflecting morphological apoptotic changes (Figure 6A). The influence of 100 μM H_2_O_2_ causes both cytoplasmic and nuclear localization of CellROX^®^ fluorescent signal, while all cells had nuclear localization of a bright green fluorescent signal during exposure to 500 μM H_2_O_2_. ApoA-I reduced the concentration of ROS, prevented nuclear localization of signal and preserved of the normal morphology of cells when it had been used in suitable concentration (obtained by cell cycle analysis), specifically 20 µg/mL for 100 μM H_2_O_2_ and 80 µg/mL for 500 μM H_2_O_2_ (Figure 6A).

The fluorescence intensity of intracellular CellROX^®^ was 1134.3 ± 22.4 and 1635.4 ± 53.3 after addition of 100 μM and 500 μM H_2_O_2_, respectively. The apoA-I significantly reduced the fluorescence intensity to 49.9 ± 0.5 and 149.7 ± 16.8, respectively (Figure 6B).

Thus, apoA-I decreased intracellular ROS levels in a dose-dependent manner during exposition of 100 and 500 µM H_2_O_2_ (Figure 6A,B).

Thus, the antioxidant potential of apoA-I has been tested under extreme conditions, such as serum deficiency and H_2_O_2_-induced oxidative stress. We showed that apoA-I was able to reduce intracellular ROS levels significantly, thereby supporting high cell proliferative rate and elevated living cell content under such conditions.

### 2.5. ApoA-I Protects MSCs under Changing Conditions

A high level of external ROS is crucial for MSCs at the exit from dormancy stage, and it is important to protect cells from damage caused by ROS. The oxygenation leads to an increase of ROS level and may enhance cell apoptosis. Therefore, we performed precondition by SD and hypoxia (24 h) followed by oxygenation (21% O_2_) with or without of apoA-I (20 µg/mL) supplementation. Results of flow cytometry showed no down regulation of cell cycle progression in case of high initial cell proliferation rate (MSCs of 22 passages) in SD and hypoxia cultivation conditions (Supplementary Figure S2). These MSCs underwent an increase of apoptosis (4.8 ± 0.3% up to 6.4 ± 0.5%), including a decrease of the total number of cells after they were transferred into oxygenation condition for 3 h (Supplementary Figure S2B). While, apoA-I induces the elevation of the S and G2/M phases (17 ± 1.2% and 23 ± 1.9%) and decreases the number of apoptotic cells to 3.8 ± 0.2%, thereby significantly increasing total cell count by 2.5 times in the same conditions (Supplementary Figure S2C).

Precondition by SD/hypoxia in presence of apoA-I protects MSCs against subsequent oxidative stress (100 µM H_2_O_2_) (Supplementary Figure S3).

### 2.6. Blood Plasma Samples of T2D Patient Influence MSCs Proliferation Rate and Survival in a Negative Manner

**Figure 7:**
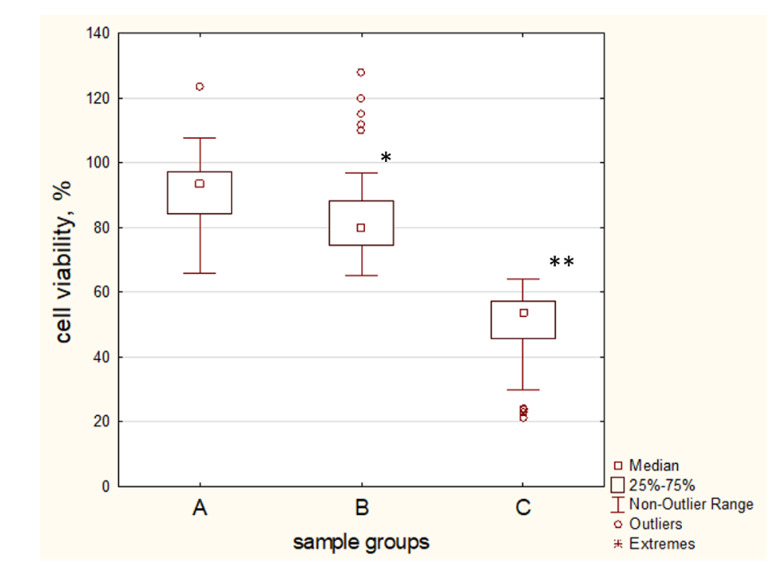
The effect of plasma samples of type 2 diabetes (T2D) patients on the viability of MSCs was assayed by MTT analysis. A—MSCs was treated by 5% *v*/*v* plasma samples of healthy donors; B, C—MSCs was treated by 5% *v*/*v* plasma samples of T2D patients. Groups were divided by a cell viability threshold of 60%. Group B—MSCs viability above 60%, group C—MSCs viability ≤ 60% and attend by cell death ≥ 10%. Group survival was calculated compared with control cells incubated in a complete nutrient medium and taken as 100%. * *p* < 0.05, ** *p* < 0.01 compared with control.

The effectiveness of MSCs transplantation in T2D was shown in 40% of cases only. The effect of the blood plasma of T2D patients on MSCs survival was evaluated by the MTT assay analysis. Our data demonstrated that the survival rate of the cells, when exposed to the blood plasma of healthy donors, was 99.22% (72.1–123.7%) (Figure 7). It should be noted that 39% of plasma samples of 89 T2D patients studied had a toxic effect on MSCs, reducing cell viability to 49.4% (21.3–63.3%), increasing the number of dead cells by more than 10%. The main pool of plasma samples of T2D (61%) reduced the proliferative activity of MSCs, cell survival was 80.8% (68.9–97%).

### 2.7. Patient T2D Blood Plasma Samples Have a High Variability of Antioxidant Protection against H_2_O_2_-Induced Oxidation

The blood plasma contains a lot of antioxidants (ascorbate, urate, ceruloplasmin, transferrin, proteins containing SH-groups, protein-lipid complexes), which provide protection against ROS. It has been shown that, in the case of T2D, the level of ROS is elevated, and thereby the estimation of the blood plasma of T2D patients to neutralize the elevated level of ROS (the antioxidant activity) is very important.

**Figure 8:**
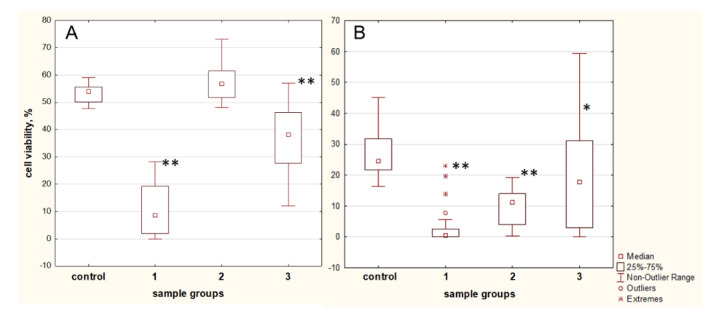
Antioxidant properties of blood plasma samples of T2D patients assayed by the MTT assay on the H_2_O_2_ oxidative stress model. (**A**) 5% *v*/*v* and (**B**) 0.5% *v*/*v* of plasma samples added to MSCs culture media before 100 µM H_2_O_2_ treatment for 24 h. Abbreviations: Plasmas of healthy donors are presented as a control group; 1, 2, 3—plasma samples of T2D patients. 1—low antioxidant activity, cell viability less than 25%; 2—elevated antioxidant activity compared with control group; 3—antioxidant activity, cell viability in the range of 25–50%. Data are shown as median (interquartile range). * *p* < 0.05, ** *p* < 0.01 compared with control group.

The blood plasma of healthy donors, when added to the culture medium (5% *v*/*v*), promoted survival of 53.9% (50.3–55.6%) cells under the conditions of 100 µM H_2_O_2_ for 24 h (Figure 8A, control).

Analysis of the antioxidant properties of the blood plasma of T2D patients showed that 45.2% of samples have very low cell survival values (8.5% (2–19.3%)) (Figure 8A, group 1). It should be noted that 16.1% of plasma samples contributed to the survival rate of 56.7% (51.7–60.5%) which were equal of healthy donors (Figure 8A, group 2). Group 3 contains 38.7% of the samples and contributes to the survival of 38.1% (28.5–45.4%) of the cells (Figure 8A, group 3).

Statistically significant differences were observed between all study T2D groups (Figure 6A). Thus, the antioxidant activity of the blood plasma of T2D patients shows significant fluctuations between individuals.

It was noted that the low concentrations of healthy donor plasmas (0.5% *v*/*v*) also had a protective effect on the MSCs viability (to 24.4% (22.8–31.2%)) under peroxidation conditions (Figure 8B, control). Cell viability was very low 0.5% (0–2.5%) when plasmas of group 1 (Figure 8A) was added to culture medium at a concentration of 0.5% *v*/*v* (Figure 8B, group 1). Interestingly, the group of high antioxidant properties (Figure 8A, group 2) did not retain the high protective abilities when was added to culture medium at a concentration of 0.5% *v*/*v*. The cell viability was 11.0% (4.0–14.0%) (Figure 8B, group 2). The samples from group 3 supported the viability of the cells in the boundaries 17.9% (2.9–31.2%) (Figure 8B, group 3).

Thus, 81.3% of the plasmas did not retain their antioxidant properties when were diluted to 0.5% *v*/*v* compared with samples of healthy donor.

### 2.8. The Influence of apoA-I on Antioxidant Properties of T2D Patient’s Plasmas

Taking into account the ability of apoA-I to protect MSCs under H_2_O_2_-induced oxidation, we wondered whether apoA-I addition to improve the reduced antioxidant properties of blood plasmas of T2D patients in the conditions of oxidation. ApoA-I (20 µg/mL) elevates MSCs viability to 35.3 ± 2.8% under conditions of 100 µM H_2_O_2_ for 24 h, as was shown by MTT test.

Toxic plasmas of T2D patients group 1 did not have antioxidant property when added to culture medium at 5% *v*/*v* (Figure 8A, group 1), the addition of apoA-I increased cell viability insignificantly in 18.2% of cases. The increased antioxidant activity was achieved by pre-incubation with apoA-I when plasmas were diluted to 0.5%. Elevated cell viability by 17.8% (10–30.1%) was obtained in 83.3% samples.

ApoA-I did not influence the antioxidant activity of plasmas of group 2 (Figure 8A) which has high activity on its own. In this group, apoA-I increases cell viability by 23% (11.2–33.5%) in 35% cases when plasmas were diluted.

Meanwhile, the apoA-I addition to plasmas of T2D patients of group 3 (Figure 8A) ameliorated cell viability both 5% *v*/*v* and 0.5% *v*/*v* of plasmas at 44% and 65% cases respectively. Since this group has enough antioxidant activity at 5% *v*/*v*, apoA-I increases of MSCs viability by 13.7% (8–19.3%) only. The dilution of plasmas results in wide range of antioxidant activity, including low level, and in this case, apoA-I increased cell viability by 26% (19.0–29.7%).

## 3. Discussion

The functional activity of the apoA-I depends on the high content of α-helices [49,50]. The oxidative modifications of the protein and/or wrongly chosen conditions of its isolation change the conformational structure of protein, increasing the content of disordered structures in the Amid I band. This leads to the appearance of cell proliferation inhibition properties [51] and a distortion of anti-inflammatory and antioxidant properties of apoA-I [52]. In this work, we studied the effect of apoA-I with a high content of α-helices on the proliferation and viability of MSCs under the stressful effects of serum and oxygen deprivation and increased ROS level in order to use the protein as an agent to increase the efficiency of MSCs transplantation.

Similar to stem cell niches environmental conditions, the serum and oxygen deprivation (5% O_2_) was accompanied by the transition of MSCs to the phase of the G0/G1 cell cycle (78.7 ± 2.1%), a decrease of Mg^2+^-dependent nuclease enzymatic activity at all pH values, and the absence of apoptotic events. Importantly, our studies demonstrate that the addition of apoA-I did not cause MSCs to re-enter the cell-cycle for proliferation under these conditions. Data on the effect of SD and hypoxia on the survival of MSCs are contradictory. The authors [53] demonstrated the growth of caspase dependent on apoptosis, while in a study by Cheung at al. [54], the transition of cells to the stage G0/G1 was noted. In our work, we noted that the degree of cell apoptosis significantly depends on the level of MSCs proliferation under stressful conditions. In the case initial high cell proliferation (S + G2/M ≥ 50%), there are insignificant decrease of proliferative activity, which is accompanied by an increase of apoptosis. ApoA-I insignificant inhibits proliferative activity additionally, and protects MSCs against apoptosis in the case of high initial proliferative activity of the cells undergoing SD/hypoxic conditions.

Cells are protected from apoptotic death by the decrease of acidic apoptotic nucleases activity under conditions of acidification of the medium during ischemia, hypoxia, and the formation of ROS [55]. Hif-1α inducible endonuclease G cleaves chromatin DNA into nucleosomal fragments independently of caspases under alkaline pH [56]. It was shown that a mimetic of apoA-I reduces the expression of Hif-1α [57] and so it can inhibit the activity of endonuclease G. Since the supplementation of apoA-I leads to additional inhibition of the activity of both acidic apoptosis and alkaline nucleases, stronger cells protect against the apoptosis under severe microenvironment conditions.

ApoA-I stimulated MSCs proliferation under SD conditions, but at normoxia only. It is known that serum deprivation induces the exit of early G1 cells from the cell cycle, which leads them to enter a quiescent state [54]. It is possible that apoA-I stimulates the proliferation of cells that have passed the R point of the G1 phase of the cell cycle, protecting dividing cells from apoptosis and thereby increasing the total number of viable cells under the conditions of serum deprivation. Therefore, apoA-I may be an important component of serum free medium, because it does not impair the quiescent state, stimulates proliferation, and protects cells from apoptosis under SD conditions.

H_2_O_2_ is an activator of MSCs proliferation; on the other hand, it has a genotoxic effect at the high concentrations. It is noteworthy that apoA-I reduces the ROS level under conditions of H_2_O_2_ oxidation and maintains high proliferative potential, protecting MSCs from apoptosis and doubling the number of viable cells compared to the control samples. A high external ROS level is critical to MSCs at the stage of recovery from dormancy; excessive accumulation of intracellular ROS is accompanied by depletion of antioxidant systems, causing aging and apoptosis of stem cells [58]. Strategies aimed at preventing the accumulation of ROS and maintaining the equilibrium of the dormant state of MSCs are urgently needed in order to increase the effectiveness of MSCs therapy. It seems that apoA-I is suitable for this task. Various conditions for the preconditioning of MSCs, including hypoxia [59], are widely used to improve the efficiency of cell transplantation. Our results demonstrate that the pre-incubation by SD/hypoxia and subsequent transfer of cells under the condition of oxygenation or the condition of oxidation stimulate their proliferation and increased apoptosis events. The addition of apoA-I supported or stimulated proliferation, reducing apoptosis and increasing the total number of cells under such conditions (Supplementary Figures S2 and S3).

The critical importance of apoA-I concentration under oxidative stress should be noted. A low amount of apoA-I with a high content of ROS leads to elevated cell death compared with control samples. Reduced apoA-I concentration in atherosclerosis, metabolic syndrome and T2D worsens the course of these diseases, complicating their cardiological component [29,60]. The same diseases are complicated by oxidative modifications, carbamylation, and nitrosylation of the protein. Recently, reactive halogen species and their role in the oxidation of proteins and LDL particles have also been investigated [61]. These modifications significantly change the functional activity of apoA-I, and in this case, there is no correlation of high levels of HDL/apoA-I with a better outcome of the disease [29,40,41,60].

Various risk factors associated with T2D, including obesity, hypertension, cardiovascular disease, and atherosclerosis, are associated with an inflammatory condition and increased cellular aging [23,62]. The efficiency of cell therapy is affected not only by internal metabolic disturbances of the applied stem cells (long propagation for large amount of MSCs, the source of cells), but also by concomitant metabolic dysregulation with oxidative stress of the recipient [63]. Diabetes is characterized by a state of increased oxidative stress. However, the published data about impaired antioxidant defense in patients with diabetes are contradictory [64].

Our data demonstrated a wide range of antioxidant responses to H_2_O_2_-induced oxidative stress and a decrease in the volumetric antioxidant defense capacity of the blood plasma of T2D patients in 81.3% of cases compared to healthy individuals. The reduced antioxidant capacity of plasma samples from T2D patients most likely implies less adaptability to changes in the microenvironment caused by inflammation.

Furthermore, 39% of the analyzed plasma samples have a cytotoxic effect on MSCs. It can be assumed that MSC-therapy will be less effective for such patients. Earlier, we showed that platelet lysate rich plasma of healthy donors activates the proliferation of MSCs and human fibroblasts [65,66]. Other authors proposed the use of platelet rich plasma for the cultivation of MSCs [67,68]. Our results suggest that incubation of MSCs in autologous plasma can adversely affect their viability and stemness.

The results of the positive effect of apoA-I in an in vitro system on MSCs were unequivocal. The response of cells to apoA-I in the model system containing plasma samples was not so straightforward. Thus, the addition of apoA-I to toxic plasma samples increased their antioxidant abilities in only 18.2% of cases. The best results were achieved when apoA-I were added to plasmas with low antioxidant property and without strong toxicity.

Clinical trials demonstrated high effectiveness transplantation of MSCs in T2D in 40% of recipients only [9,20]. The limited number of successful treatments can be explained, among other things, by the results of a study on the effect of blood plasma on MSCs viability. The state of the HDL/apoA-I system, both of the donor and the recipient, can have both positive and negative effects on the success of the transplantation, and it can be taken into account and adjusted.

The unique properties of apoA-I protein stimulated considerable interest in studying both the native protein and the preparation of its mimetics with an amphipathic a-helical structure similar to the secondary structure of the a-helical apoA-I region. Currently, clinical trials with positive results have been conducted [46]. It is important to note that apoA-I stimulates insulin secretion by beta cells and enhances insulin-dependent and insulin-independent glucose uptake and so improves glycemic control and attenuate insulin resistance of T2D patients [36,69,70].

Based on the foregoing, it can be assumed that not only incubation of MSCs with apoA-I in serum-free medium may be useful for the propagation and maintenance of stem cells. MSCs transplantation in the presence of an additional amount of fully functional apoA-I and/or its mimetics may be more effective for the treatment of T2D patients and its complication.

## 4. Materials and Methods

### 4.1. Cell Culture

In this work, we used rat and human bone marrow-derived mesenchymal stem cells (MSCs). Human MSCs 4–6 passage were taken from the culture bank of Research Institute of Clinical and Experimental Lymphology—Branch of the ICG SB RAS (RICEL–branch ICG SB RAS). Rat MSCs were harvested from the femoral and tibia bones of Wistar rats (8 weeks old) according to standard protocol. Cells in the fourth passage were used for phenotype screening of MSCs by flow cytometry (CytoFLEX S, Beckman Coulter, Brea, CA, USA) using antibodies to CD90FITS, CD90APC, CD29APC, CD73FITS (BD Biosciences, San Jose, CA, USA), and hematopoietic cell marker CD45FITS (BD Biosciences, San Jose, CA, USA).

All animal procedures were conducted in accordance with ethical standards of the international, institutional animal care guidelines. The Committee of Ethics in Animal Research of the Federal Research Center of Fundamental and Translational Medicine reviewed and approved all animal procedures (No. 10 of 26/03/2019).

### 4.2. Blood Plasma Samples of T2D Patients

The study involved 89 T2D patients. Exclusion criteria were severe concomitant pathology, skin infectious diseases in the acute stage, acute inflammatory processes, pregnancy and lactation, and malignant neoplasms. The control group consisted of 15 age-matched men and women who did not have a verified diagnosis of T2D, who did not smoke or drink alcohol for at least a week before blood samples taking. Blood plasma containing platelet lysate samples was prepared as previously described with slight modifications [65]. Plasma was collected and stored at −70 °C until use. The study protocol was approved by the local ethics committee on 12 October 2017 (protocol No. 135). The informed consent of the patient to the examination was taken in accordance with the directives of the European Community (86/609/EEC) and the Helsinki Declaration, in compliance with the Ethical Principles for Scientific Medical Research with Human Participation and in accordance with the Rules of Clinical Practice in Russian Federation.

### 4.3. ApoA-I Isolation and Characterization of Its Secondary Structure

The apoA-I was isolated from healthy blood donors by the method of sequential isolation of high density lipoproteins (HDL) by isodensity ultracentrifugation in KBr solutions (Optima L-90K, Beckman Coulter, Brea, CA, USA) followed by delipidization of HDL with a mixture of butanol-diisopropyl ether in a ratio of 1: 3 and subsequent gel filtration (Sephacryl, Sigma-Aldrich, St. Louis, MO, USA). The purified apoA-I were analyzed by electrophoresis on 15% SDS-PAGE followed by gel densitometry of the bands. Standard molecular weight markers (10–80 kDa) (Thermo Fisher Scientific, Waltham, MA, USA) were used.

The secondary structure of the protein was determined using IR spectrometry. Briefly, the Fourier Transformed Infrared (FTIR) spectra were recorded using Nicolet 6700 spectrometer (Thermo Fisher Scientific, Waltham, MA, USA) in Attentual Total Reflection (ATR) mode by using Smart Orbit Diamond ATR. For the quantification of the ratio of protein secondary structure, the solution of the apoA-I in phosphate-buffered saline (PBS) (10 µL) with the mass of the protein of 7.5 µg was spread on the diamond crystal. The spectra were collected three times. The number of scans was set to 100 and the resolution of spectra were 4 cm^−1^. For each measurement, the Amide I band in the range from 1600 to 1700 cm^−1^ was deconvoluted and the second derivative was estimated using OriginPro software (OriginLab Corporation, Northampton, MA, USA) after subtraction of the spectrum of PBS. The fitting of the Amide I band was performed in Origin Pro software as described in [71]. The quantification of α-helix, β-strand, turns, and the disordered structure was performed for each measurement and the mean values with standard deviations are reported in the results.

### 4.4. Cultivation Conditions

Cells were cultured in Iscove’s Modified Dulbecco’s Medium (IMDM, Gibco, Carlsbad, CA, USA) supplimened with 10% fetal bovine serum (FBS, Gibco, Carlsbad, CA, USA) and 50 μg/mL gentamicin (Gibco, Carlsbad, CA, USA) under standard cultivation condition (37 °C, 5% CO_2_, 20% O_2_ at humidified atmosphere) and were used as control.

### 4.5. Serum Deprivation

MSCs were seeded in completed media for 24 h followed by refreshed medium without FBS.

### 4.6. Hypoxic Conditions

MSCs were cultivated in growth medium IMDM under hypoxic conditions (37 °C and 5% CO_2_, 5% O_2_ at humidified atmosphere) in a sealed chamber. The oxygen concentration in the medium was measured before and after the experiment by Clark’s portable electrode. The proper pH volume of the culture medium was adjusted by adding HEPES buffer (10 mM).

### 4.7. Oxidative Stress Conditions

Cells were incubated in a culture medium containing 100 or 500 µM H_2_O_2_ for 2 (for intracellular ROS detection) or 6 and 24 h (for the detection of cell viability, cell cycler analysis, and antioxidant property of plasmas samples).

### 4.8. Plasmid Nicking Assay of DNase Activity

The nuclease activity of MSCs was analyzed by agarose gel electrophoresis using as a substrate plasmid DNA Bluescript (pDNA). The MSCs were cultured in culture plates at a density of 1 × 10^6^ cells/500 μL/well. The experimental samples were cultured under conditions of serum deprivation, as well as in conditions of normoxia or hypoxia. ApoA-I was added to the culture medium at concentration of 20 μg/mL. The level of nuclease activity was determined in the culture medium and in the cell homogenate. Each test sample (5 μL) was incubated at 37 °C in 20-μL nuclease reaction buffer (50 mM Tris-HCl [pH 8.3 or 7.2 or 5.5] and 50 mM MgSO4) containing 2 μg of pDNA. The negative control was prepared using 2 µg of pDNA in 5 µL of water and employed using the same incubation conditions. Further, ethylenediaminetetraacetic acid (EDTA) was added to each probe, after 40 min reaction. The reaction products were analyzed by 0.8% agarose gel electrophoresis and visualized by staining with ethidium bromide. Supercoiled, nicked, and linearized pDNA were quantified with the ImageJ software. The measurements were carried out three times in 3 independent experiments.

### 4.9. MTT Assay

MSCs were cultivated in 96-well culture plates at a density of 7 × 10^3^ cells/100 μL/well. The samples were cultured under conditions of serum deprivation, as well as in conditions of normoxia or hypoxia, with or without apoA-I to detect it influence on MSCs viability.

Subsequently, 5% *v*/*v* of plasma samples were added to cells for the detection of cytotoxicity of plasma samples of T2D patients. Cell viability less than 60% compared with control was clarified as toxic sample.

The antioxidant activity of blood plasma was determined using 100 µM H_2_O_2_ for 24 h. The MSCs were cultured in the presence of the 5 or 0.5% *v*/*v* patients serum and 5% FBS. Control cells were incubated in present 10% FBS and 100 µM H_2_O_2_. 10 μL of the MTT solution (5 mg·mL^−1^) was added to each well, and the plates were incubated for a further 4 h. The formazan produced was then dissolved in dimethyl sulfoxide (DMSO) (100 μL). The optical density of the solutions was measured by Multiskan FC spectrophotometer (Thermo Fisher Scientific, Waltham, MA, USA) at 570 nm. Relative cell viability (%) was expressed as a percentage of control cells taken as 100%. Plasmas of T2D patients protecting cells viability by less than 50% from control level was considered as a sample with a low antioxidant activity.

### 4.10. 3H-Thymidine Incorporation Assay

The MSCs were cultured in 24-well culture plates at a density of 5 × 10^5^ cells/500 μL in SD condition for 6, 18, and 24 h. ApoA-I was added to the test samples at a concentration of 20 μg/mL. 3H-thymidine (2.0 µCi/mL) was added 2 h before cell harvesting. The radioactivity of the samples was measured on a Scintillation System LS 6500 (Beckman Coulter, Brea, CA, USA). Speed of label inclusion was calculated in pulses of 1 min per 10^6^ cells. All measurements were performed in triplicate in three independent experiments

### 4.11. Cell Cycle Analysis

MSCs were cultivated in 6-well culture plates at a density of 50 × 10^4^ cells/500 μL/well under SD, hypoxia and oxidative stress conditions in the presence or absent of the apoA-I for 6 and 24 h. Cell cycle were estimated using Propidium Iodide (PI) staining and analyzed by CytoFlex (Backman Coulter, Brea, CA, USA) λEm = 670 nm. Data were analyzed on Kaluza software (Backman Coulter, Brea, CA, USA).

### 4.12. Detection of Reactive Oxygen Species (ROS) by CellROX Reagent

Cellular oxidative stress was detected using the cell-permeable CellROX^®^ Green Reagent (Thermo Fisher Scientific, Waltham, MA, USA) according to the manufacturer’s instructions. MSCs were seeded in 96 well plates in concentration of 7 × 10^3^ cells/well. ApoA-I was added in culture medium 10 min before 100 and 500 µM of H_2_O_2_ was added for 2 h. Cells were observed using fluorescence microscopy (Zeiss, Axio observer Z1, Jena, Germany) at 545 nm excitation and 565 nm emission wavelength and analyzed using Cell Activision R1.03.01 software (Yokogawa Electrics Corporation, Tokyo, Japan). Results were expressed as fluorescence nominal units.

### 4.13. Statistical Analysis

In the present study, STATISTICA 7.0 (StatSoft, Inc., Tulsa, OK, USA) was used for statistical analyses. The normality of the data was evaluated through the Kolmogorov–Smirnov test. Normally distributed variables were expressed by mean ± standard deviation (SD) and significant differences between groups were analysed by Student’s *t*-test. Parameters that were not normally distributed were expressed by median ± interquartile range (IQR) and significant differences between groups were analyzed by the Mann–Whitney U test.

## 5. Conclusions

ApoA-I supports normal cell homeostasis by influencing MSCs proliferation within the physiological response of cells by the absence of the activation of proliferation under niche conditions, contact inhibition, and high proliferation rate. ApoA-I supports increased cell proliferation and protects cells from apoptosis in conditions of oxidative stress and serum deprivation, thus enlarging the pool of viable cells. Preconditioning MSCs with the protein under SD and hypoxia elevated proliferative rate, decreasing apoptosis during cell transition under conditions of oxygenation and oxidation.

Individual plasmas of T2D patients were found to have high variability of antioxidant activity. It was found that 81.3% of blood plasmas have low volume antioxidant defense and 39% of plasmas have high cytotoxicity effect on MSCs. It is known that apoA-I can stimulate insulin secretion and glucose uptake, improving the control of glycemic levels of T2D patients. These unique properties of the protein, together with our findings of its effect on MSCs, suggest great potential for the development of regulatory MSC/apoA-I-mediated approaches for the treatment of T2D patients.

## Supplementary Materials

Supplementary materials can be found at https://www.mdpi.com/1422-0067/21/11/4062/s1, Figure S1: Full 0.8% agarose gels electrophoregram illustrating the intracellular and secreted nuclease activity, Figure S2: Cell cycle of MSCs cultivated under SD and hypoxia conditions for 24 h (precondition) (**A**) followed by 3 h under normoxic condition (**B**) and 3 h normoxic condition with apoA-I (**C**), Figure S3: Cell cycle of MSCs cultivated under SD and hypoxia conditions for 24 h (precondition) followed by (**A**) 3 h under normoxic condition with addition of H_2_O_2_ 100 µM; (**B**) 3 h normoxic condition with addition of apoA-I and H_2_O_2_ 100 µM (**B**) and (**C**) MSCs cultivated under SD and hypoxia conditions with apoA-I for 24 h followed by 3 h normoxic condition with addition of apoA-I and H_2_O_2_ 100 µM.

## Abbreviations

MSCs	Mesenchymal Stem/Stromal Cells
ApoA-I	Apolipoprotein A-I
HDL	High-Density Lipoprotein
LDL	Low-Density Lipoprotein
ROS	Reactive Oxygen Species
SD	Serum Deprivation
DNA	Deoxyribonucleic Acid
FBS	Fetal Bovine Serum
SDS-PAGE	Sodium Dodecyl Sulfate–Polyacrylamide Gel Electrophoresis
FTIR	Fourier Transform Infrared Spectroscopy
