# Supplementary material for: Apolipoprotein A-I Supports MSCs Survival under Stress Conditions
Source: Int J Mol Sci. 2020 Jun 5;21(11):4062. doi: 10.3390/ijms21114062 (PMC7312015; doi:10.3390/ijms21114062)
Supplement: Supplementary file 1 [file ijms-21-04062-s001.pdf]

International Journal of
Molecular Sciences

MDPI

Supplementary Materials

ApolipoproteinA-1 Supports MSCs Survival under Stress Condition

Svetlana Miroshnichenko [1,2]*, Ivan Usynin [1], Alexey Dudarev [1], Vadim Nimaev [2] and Anastasiya Solovieva [2]*

[1] Institute of Biochemistry, FRC FTM, 2 Timakova str., 630117 Novosibirsk, Russian Federation, svmiro@yandex.ru (S.M.),

[2] Research Institute of Clinical and Experimental Lymphology– Branch of the ICG SB RAS, 2 Timakova str., 630060 Novosibirsk, Russian Federation, svmiro@yandex.ru (S.M.), solovevaao@gmail.com (A.S.), nimaev@gmail.com (V.N.)

* Correspondence: svmiro@yandex.ru, solovevaao@gmail.com

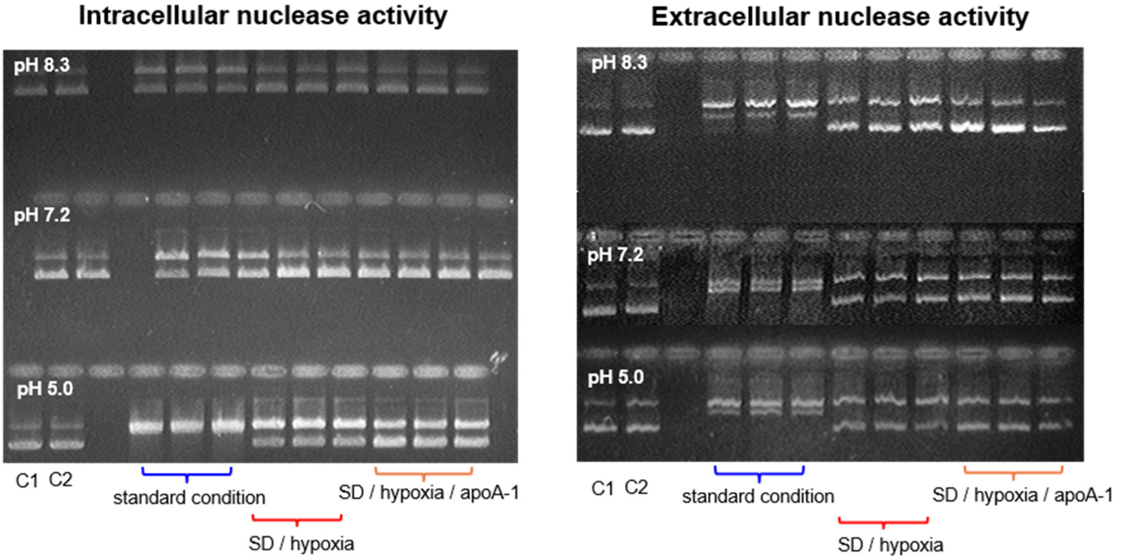

Figure S1. Full 0.8% agarose gels electrophoregram illustrating the intracellular and secreted nuclease activity. C1—control 1-hole pDNA, C2—control 2-pDNA/cells (for intracellular) or media (for secreted)/EDTA.

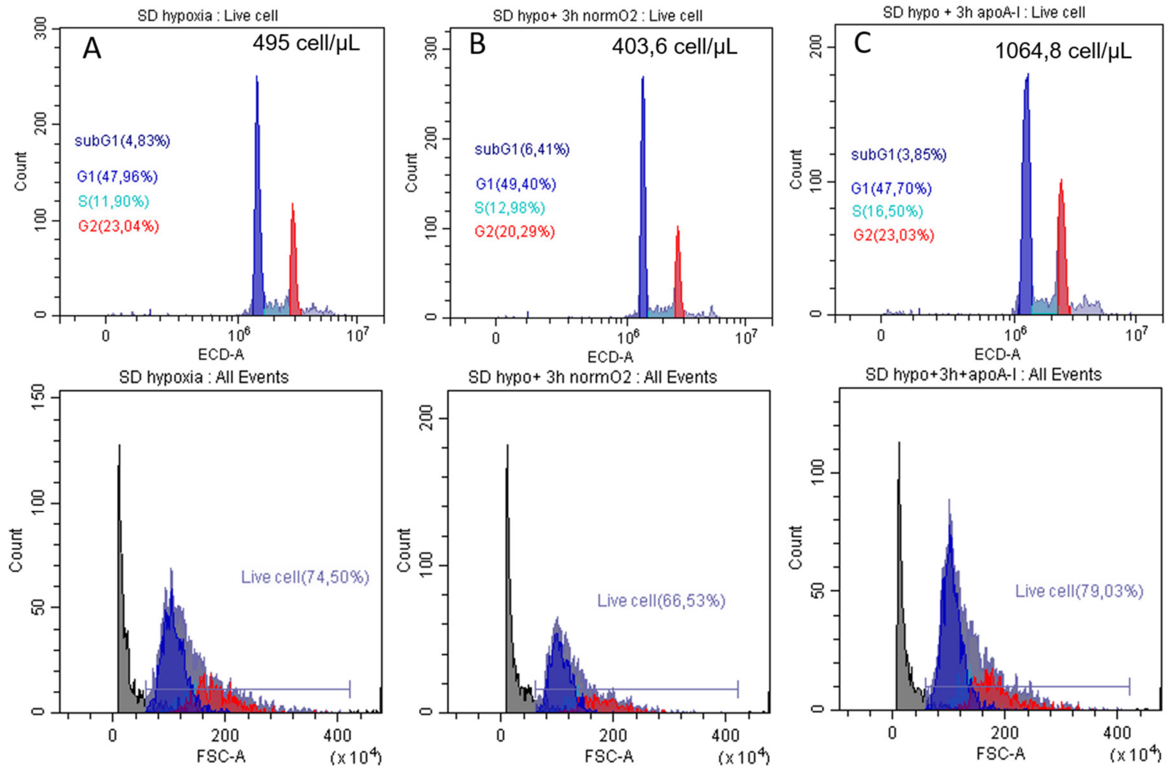

Figure S2. Cell cycle of MSCs cultivated under SD and hypoxia conditions for 24 hours (precondition) (**A**) followed by 3 hours under normoxic condition (**B**) and 3 hours normoxic condition with apoA-I (**C**).

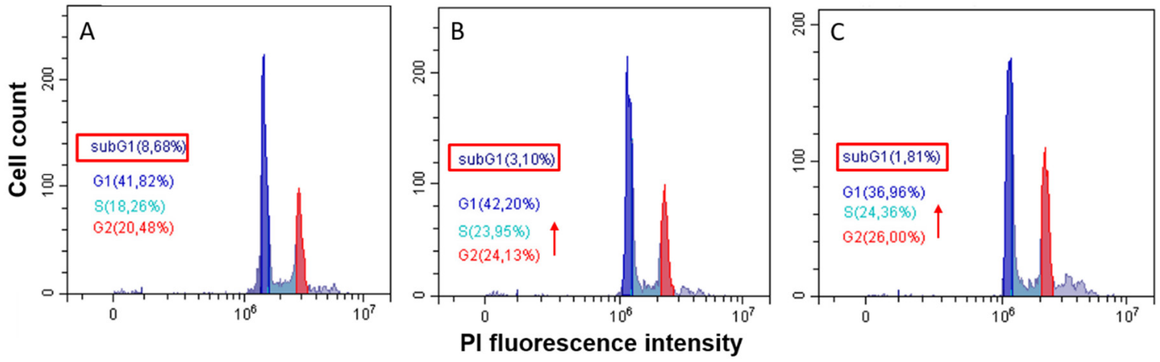

Figure S3. Cell cycle of MSCs cultivated under SD and hypoxia conditions for 24 hours (precondition) followed by (**A**) 3 hours under normoxic condition with addition of H_2O_2 100 µM; (**B**) 3 hours normoxic condition with addition of apoA-I and H_2O_2 100 µM (**B**) and (**C**) MSCs cultivated under SD and hypoxia conditions with apoA-I for 24 hours followed by 3 hours normoxic condition with addition of apoA-I and H_2O_2 100 µM.

© 2020 by the authors. Submitted for possible open access publication under the terms and conditions of the Creative Commons Attribution (CC BY) license (http://creativecommons.org/licenses/by/4.0/).
